# Effects of the hydroalcoholic extract of *Rosa damascena* on hippocampal long-term potentiation in rats fed high-fat diet

**DOI:** 10.1186/s12576-021-00797-y

**Published:** 2021-04-29

**Authors:** Seyed Asaad Karimi, Somayeh Komaki, Masoumeh Taheri, Ghazaleh Omidi, Masoumeh Kourosh-Arami, Iraj Salehi, Alireza Komaki

**Affiliations:** 1 Neurophysiology Research Center, Hamadan University of Medical Sciences, Hamadan, Iran; 2 Department of Neuroscience, School of Science and Advanced Technologies in Medicine, Hamadan University of Medical Sciences, Hamadan, Iran; 3 Department of Neuroscience, School of Advanced Technologies in Medicine, Iran University of Medical Sciences, Tehran, Iran; 4 Neuroscience Research Center, Iran University of Medical Sciences, Tehran, Iran; 5 Department of Physiology, School of Medicine, Hamadan University of Medical Sciences, Shahid Fahmideh Street, 65178/518 Hamadan, Iran

**Keywords:** *Rosa damascena*, Long-term potentiation, Hippocampus, High-fat diet, Antioxidant, Synaptic plasticity

## Abstract

High-fat diets (HFDs) and obesity can cause serious health problems, such as neurodegenerative diseases and cognitive impairments. Consumption of HFD is associated with reduction in hippocampal synaptic plasticity. *Rosa damascena* (*R. damascena*) is traditionally used as a dietary supplement for many disorders. This study was carried out to determine the beneficial effect of hydroalcoholic extract of *R. damascena* on in vivo hippocampal synaptic plasticity (long-term potentiation, LTP) in the perforant pathway (PP)—dentate gyrus (DG) pathway in rats fed with an HFD. Male Wistar rats were randomly assigned to four groups: Control, *R. damascena* extract (1 g/kg bw daily for 30 days), HFD (for 90 days) and HFD + extract. The population spike (PS) amplitude and slope of excitatory post-synaptic potentials (EPSP) were measured in DG area in response to stimulation applied to the PP. Serum oxidative stress biomarkers [total thiol group (TTG) and superoxide dismutase (SOD)] were measured. The results showed the HFD impaired LTP induction in the PP-DG synapses. This conclusion is supported by decreased EPSP slope and PS amplitude of LTP. *R. damascena* supplementation in HFD animals enhanced EPSP slope and PS amplitude of LTP in the granular cell of DG. Consumption of HFD decreased TTG and SOD. *R. damascena* extract consumption in the HFD animals enhanced TTG and SOD. These data indicate that *R. damascena* dietary supplementation can ameliorate HFD-induced alteration of synaptic plasticity, probably through its significant antioxidant effects and activate signalling pathways, which are critical in controlling synaptic plasticity.

## Introduction

Synapses in the central nervous system (CNS) endure alterations in synaptic strength, a process called synaptic plasticity [1]. Synaptic plasticity is one of the basic mechanisms in neural circuits for most models of learning and memory [2]. These changes are collectively referred to as Hebbian plasticity, which occur locally in individual synapses and include long-term potentiation (LTP) or long-term depression (LTD) [3, 4].

Numerous studies have reported that high-fat diet (HFD) consumption can alter the morphology and structure of synapses, the amounts of released neurotransmitters and synaptic plasticity in different areas of the brain, and especially in the hippocampus [5–8]. The hippocampus is one of the brain regions critical to learning and memory [9]. Furthermore, preclinical human and rodent studies have demonstrated reduction in cognitive function and whole-body efficiency following short-term and long-term usage of an HFD [8, 10–12]. Other works have identified HFD consumption, as a risk factor for Alzheimer disease (AD) development [13].

Although the pathogenesis of synaptic plasticity and cognitive impairment following consumption of an HFD has not been fully clarified, factors, such as metabolic disorder [14], brain inflammation [15], brain insulin resistance [16], and oxidative stress [17], are believed to play probable roles.

Given the increasing universal burden of HFD consumption and obesity, an emerging issue of dementia related to HFD consumption, understanding the impacts of HFD consumption on learning and memory related mechanism(s), identification and recognizing of potential underlying mechanisms, and finding effective beneficial approaches are vital. However, until now, no effective treatments are available for the management of HFD-induced hippocampal-dependent memory and synaptic plasticity deficits. High level of inflammation and oxidative stress induced by a HFD is one of the main reasons for the reduction in synaptic plasticity [18, 19] and impaired cognitive function [20, 21]. Oxidative stress produces excessive reactive oxygen species (ROS), primarily due to imbalances in oxidative to reducing species [22, 23].

It has been shown that *Rosa damascena* (*R. damascena*) or *Damask Rose* can reduce oxidative toxicity, and has a key role in ROS disarm [24]. In our previous work, we demonstrate that treatment with the hydroalcoholic extract of *R. damascena* can prevent cognitive impairment caused by the consumption of an HFD, as measured by the passive avoidance learning test [25]. Since LTP is one of the basic mechanisms for learning and memory, in this study, we examined the effects of *R. damascena* on LTP.

*R. damascena* known as “Gole Mohammadi” in Iran is one of the most important species of Rosaceae family flowers [26]. *R. damascena* is one of the most famous ornamental plants in herbal medicine and is used as a traditional remedy in Iran [26] because of its sedative [27], anti-inflammatory [28, 29], antibacterial [30], analgesic [31], antioxidant [30, 32], anticancer and pain relief [33, 34] effects. Extracts of this plant are also used in beauty products and perfumes in multiple countries [30, 35].

Considering the beneficial effects of *R. damascena*, here, we assessed the effect of hydroalcoholic extract of *R. damascena* on serum oxidative stress biomarkers and in vivo hippocampal synaptic plasticity (long-term potentiation, LTP) in the perforant pathway (PP)—dentate gyrus (DG) pathway in adult rats fed with an HFD.

## Methods

### Ethics statement

All experimental procedures using rats were conducted in accordance with the animal care and use guidelines approved by the institutional ethics committee at Hamadan University of Medical Sciences and were performed in accordance with the National Institutes of Health Guide for Care and Use of Laboratory Animals [36]. All efforts were made to minimize suffering. The operations that could cause pain and distress were performed in another room in the absence of other animals.

### Animals and experimental design

**Fig. 1:**
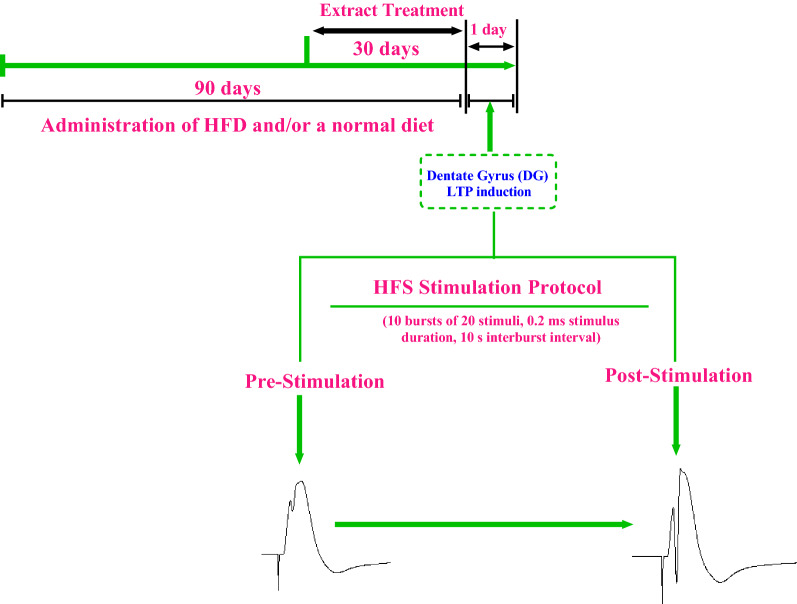
Experimental design and timeline. Animals were maintained on HFD or standard diet regimes as per the protocol for 90 days before subjecting them for electrophysiological recording. The extract (1 g/kg bw daily) was administered by oral gavage for 30 days. After that, once a stable baseline was observed for at least 40 min, LTP was induced by applying HFS consisting of 10 bursts of 20 stimuli, 0.2 ms stimulus duration, 10 s interburst interval in the DG of rats. HFS; high-frequency stimulation

Adult male Wistar rats weighing 200–250 g obtained from Pasteur Institute of Tehran, Iran. The animals were housed in an air-conditioned room at 22 ± 2 °C with a 12-h light/dark cycle. The animals were kept in cages with 2–3 rats in each cage. The animals had free access to water and standard or high-fat rat chow. Animals were randomly assigned to four groups of 6–8 animals each: (1) control (received standard diet), (2) HFD (received high-fat diet only), (3) HFD + Ext (received HFD plus 1 g/kg bw hydroalcoholic extract of *R. damascena*, (4) Standard diet + 1 g/kg bw hydroalcoholic extract of *R. damascena* (Ext group). The extract (1 g/kg bw daily) was administered by oral gavage for 1 month [25, 37, 38]. Animals were maintained on standard diet or HFD regimes as per the protocol for 11 weeks. After 90 days, LTP was induced in area DG with high-frequency stimulation (HFS). Experimental design and timeline are shown in Fig. 1.

### High-fat diet

Animals in the HFD and HFD + extract groups were fed an HFD composed of a standard diet (67.7%) with 8.3% ghee, 4.05% hydrogenated oil, 0.85% soybean oil, 0.8% sodium cholate, 1.0% cholesterol and 17.3% sugar [12, 25, 39, 40]. For concocting HFD, standard diet chows were powdered and this powder was mixed with a certain percentage of materials in the mixture and then came in the form of pellets. For feeding experiments, male rats were placed in cages and were fed with either standard diet (Behparvar, Iran) or HFD. Animals had free access to the HFD for 3 months [25]. The composition of standard diets includes 21% protein, 3.69% fat, 32.5% carbohydrates, and 5.5% raw fiber.

### Preparation of extract

For the preparation of the hydroalcoholic *R. damascena* extract, *R. damascena* petals were purchased from a flower market (Hamadan, Iran) and identified and authenticated at the Botanic Institute of the Hamadan University of Medical Sciences. These petals were dried in a room free of sunlight and then ground into a powder. This powder was then dissolved in 98% ethanol and extracted using distilled water and ethanol (1:1 v/v) as a solvent. This extract was filtered and concentrated under reduced pressure on a rotary evaporator. It was finally freeze-dried at – 80 °C. The extract was dissolved in water and prepared fresh daily in our lab and 1 g/kg bw was administered daily by oral gavage for 1 month. Doses were chosen according to previously published data [25, 31, 41–43]. The total phenolic and flavonoid content of *R. damascena* were measured by Folin–Ciocalteu reaction and colorimetric assay, respectively [25].

The median lethal dose (LD_50_) of this plant extract has been reported previously [44–47]. Oral LD_50_ of *R. damascena* and rose absolute was > 5 g/kg in rats and dermal LD_50_ of *R. damascena* was > 2.5 g/kg in rabbits [44, 47]. Consistent with results of another study, LD_50_ was determined 6 g/kg [45]. Additionally, it has been shown that an LD_50_ dose of 2 g/kg and higher than 2 g/kg is categorized as unclassified and therefore the extract is found to be safe [46].

The potential toxic results of *R. damascena* infusion in dogs at doses 90–1440 mg/kg/day (0.5–8 times of human uses) for 10 successive days discovered a minimal nephrotoxic or hepatotoxic effect. Therefore, it might also have hepatotoxic consequences at extraordinary high doses [48]. In another experiment, the ethanol extract of *R. damascena* failed to show any mortality and toxic manifestations up to the dose of 3200 mg/kg [46].

IC_50_ values of ethanol and watery extract of *R. damascena* have been discovered to be 18.46 and 22.1 lg/ml [49]. In another study, IC_50_ value of the extract in free radical scavenging was 2.24 lg/ml and in fat peroxidation assays was 520 lg/ml and fat peroxidation assays, respectively [50].

### Surgical procedure, electrophysiological recording and LTP induction

Hydroalcoholic extract of *Rosa damascena* was administered intragastrically by gavage once a day for 1 months. Then, the rats were anesthetized with urethane, and placed into a stereotaxic apparatus for surgery, electrode implantation and field potential recording. The methodologies used in this section were similar to prior studies that published by our laboratory [5, 51–54]. Briefly, under urethane anesthesia induced by intraperitoneal injection (1.5 g/kg), rats’ head was fixed in a stereotaxic apparatus for surgery and recording. A heating pad was used to maintain the temperature of the animals at 36.5 ± 0.5 °C. Small holes were drilled in the skull. Afterwards, two bipolar electrodes, made of stainless steel with Teflon cover (125 µm diameter, Advent Co., UK) were positioned in the right cerebral hemisphere. The stimulating electrode was placed in the perforant pathway (PP) [:AP: − 8.1 mm from bregma; ML: + 4.3 mm from midline; DV: 3.2 mm from the skull surface], while the recording electrode was positioned in the dentate gyrus (DG) granular cell layer [AP: − 3.8 mm from bregma; ML: + 2.3 mm from midline; DV: 2.7–3.2 mm from the skull surface] according to the Paxinos and Watson atlas of the rat brain [5, 55]. The electrodes were lowered very slowly (0.2 mm/min) from cortex to the hippocampus, to minimize trauma to the brain tissue.

Input–output current profiles were obtained by stimulating the PP to determine the stimulus intensity to be used in each animal (40% maximal population spike). Single 0.1 ms biphasic square wave pulses were delivered through constant current isolation units (A365 WPI) at a frequency of 0.1 Hz.

The field potential recordings were obtained in the granular cells of the DG following stimulation of the PP. Test stimuli were delivered to the PP every 10 s. Electrodes were positioned to elicit the maximum amplitude of population spike (PS) and field excitatory post-synaptic potentials (fEPSP). After ensuring a steady-state baseline response, which was taken about 40 min, LTP was induced using a high-frequency stimulation (HFS) protocol of 400 Hz (10 bursts of 20 stimuli, 0.2 ms stimulus duration, 10 s interburst interval) at a stimulus intensity that evoked a PS amplitude and field EPSP slope of ~ 80% of the maximum response. Both fEPSP and PS were recorded 5, 30, and 60 min after the HFS to determine any changes in the synaptic response of DG neurons. For each time point, 10 consecutive evoked responses were averaged at 10 s stimulus interval [56–58].

For stimulations, the parameters of the stimuli were defined in homemade software and were sent via a data acquisition board linked to a constant current isolator unit (A365 WPI, USA) prior delivery to the PP through the stimulus electrodes. The induced field potential response from the DG, was passed through a preamplifier, then was amplified (1000 ×) (Differential amplifier DAM 80 WPI, USA), and filtered (band pass 1 Hz to 3 kHz). This response was digitized at a sampling rate of 10 kHz, and was observable on a computer (and an oscilloscope). It was saved in a file to facilitate later offline analysis.

### Measurement of evoked potentials

The evoked field potential in the DG has two components: PS and fEPSP. During electrophysiological recordings, changes in PS amplitude and fEPSP slope were measured [5].

**Fig. 2:**
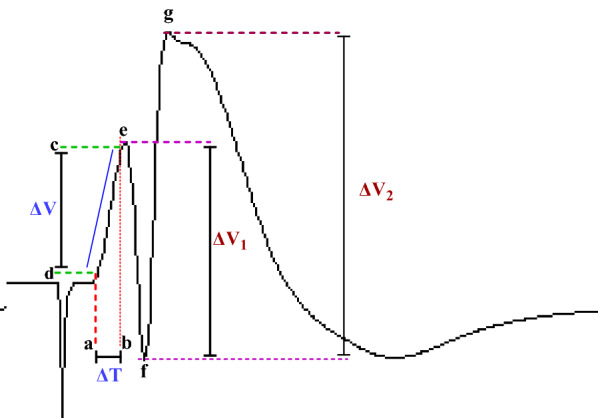
Measurement of evoked potentials. PS amplitude and EPSP slope were calculated according to Eqs. (1) and (2), respectively (refer to the text). ΔV = The potential difference, ΔT: Time difference

PS amplitude and EPSP slope were calculated according to Eqs. (1) and (2), respectively (see Fig. 2).

1 $$EPSP=\frac{\Delta V}{\Delta T},$$

2 $$PS=\frac{\Delta {V}_{1}+\Delta {V}_{2}}{2},$$

where (see Fig. 2). ΔV = the potential difference between points c and d. ΔT: Time difference between points a and b. ΔV1 = the potential difference between points e and f. ΔV2 = the potential difference between points f and g.

### Blood sampling and biochemical analyses

At the end of the study, animals were anesthetized with urethane (ethyl carbamate, 1.8 g/kg; i.p.). Blood samples were taken from the portal vein and centrifuged at 3000 rpm for 10 min at 4 °C. Plasma measurements were performed for the assay of serum oxidative stress biomarkers [total thiol groups (TTG), and superoxide dismutase (SOD)].

### Statistical analysis

Data were presented as mean ± SEM and processed by commercially available software GraphPad Prism^®^ 8.0.2. The data normality test was performed using Shapiro–Wilk test. Oxidative stress data were analyzed by one-way ANOVA and Tukey’s post hoc test. LTP data were analyzed by two-way repeated measures ANOVA followed by Bonferroni test. LTP data were normalized to the mean value of fEPSP slopes and PS amplitude recorded prior to the induction of LTP (Eq. 3) [53, 59, 60], and reported as mean ± SEM. P values < 0.05 were considered significant.

3 $${\text{LTP}} = \frac{{{\text{the}}\;{\text{EPSP}}\;{\text{or}}\;{\text{PS}}\;{\text{value}}\;{\text{after}}\;{\text{HFS}}\;{\text{induction}} \times 100\% }}{{{\text{the}}\;{\text{average}}\;{\text{EPSP}}\;{\text{or}}\;{\text{P}}\;{\text{Satbaseline}}}}.$$

## Results

### Effects of *R. damascena* extract on the biomarkers of oxidative stress in rats fed with high-fat diet

**Fig. 3:**
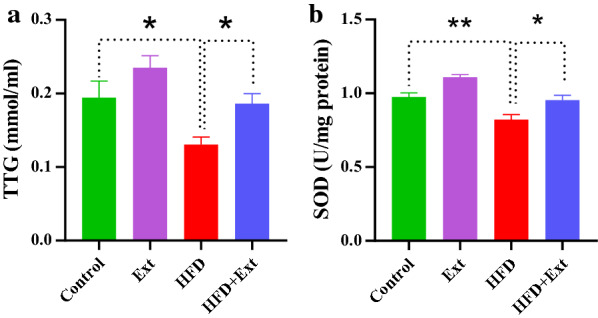
Effect of high-fat diet (HFD) and *R. damascena* extract administration on serum levels of total thiol groups (TTG) (**a**) and superoxide dismutase (SOD) (**b**). Data presented as means ± S.E.M. *p < 0.05, and **p < 0.01

**Fig. 4:**
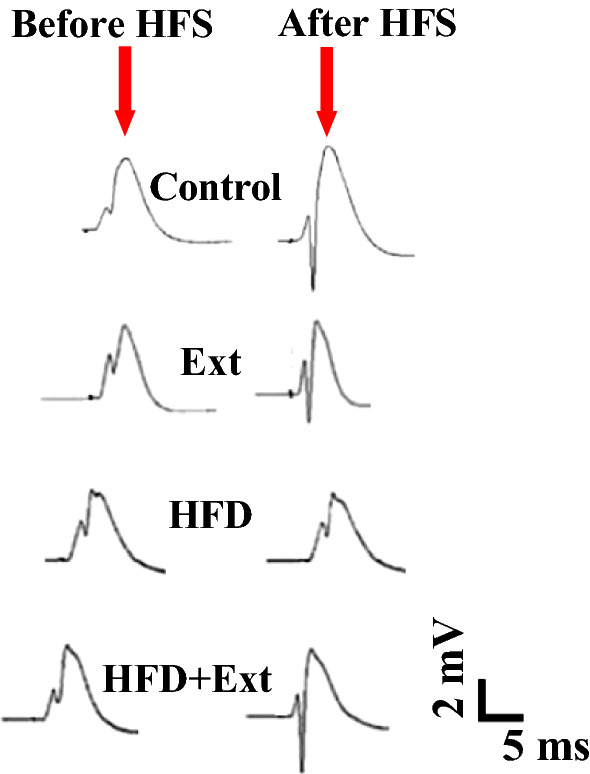
Representative sample traces of evoked field potential in the DG recorded prior to and 60 min after high-frequency stimulation in all groups

Thiol concentration increases in oxidative stress conditions [61]. The total thiol status in the body, especially thiol groups present in proteins are considered as major plasma antioxidants in vivo, and most of them are present over albumin [62], and they are the major reducing groups present in our body fluids [63]. There was a significant difference in the case TTG among the experimental groups of rats (F (3, 33) = 7.565, P = 0.0006, One-way ANOVA, Fig. 3a). As illustrated in Fig. 4a, Consumption of HFD decreased TTG in compare with control group (P = 0.0391, Fig. 3a). *R. damascena* extract consumption in the HFD group enhanced TTG (P = 0.043). Superoxide dismutases (SODs) constitute a very important antioxidant defense against oxidative stress in the body [64]. The impact of HFD and *R. damascena* extract on SOD concentration in serum was evaluated. There was a significant difference in the case of SOD among the experimental groups of rats (F (3, 32) = 18.37, P < 0.0001, One-way ANOVA, Fig. 3b). As illustrated in Fig. 3b, HFD decreased SOD in serum compared with the control group (P = 0.0045, Fig. 3b). Treatment of HFD animals with *R. damascena* extract increased SOD concentration (P = 0.0170).

### Effects of *R. damascena* extract on the field excitatory post-synaptic potential (fEPSP) slopes of granular cells in the DG of rats fed with high-fat diet

**Fig. 5:**
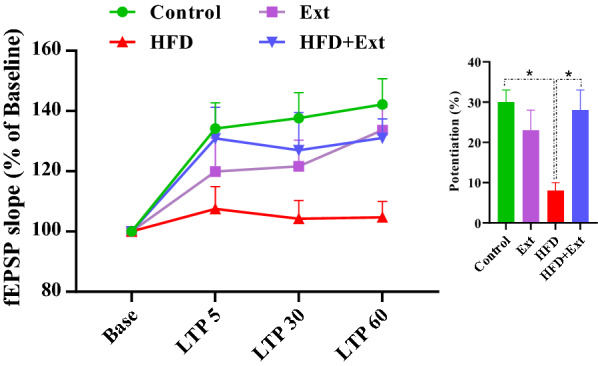
Time-dependent changes in hippocampal responses to perforant path stimulation following an HFS. LTP of the EPSP slope in area DG granular cell synapses of the hippocampus are significantly different between groups. Left panel shows fEPSP slope change (%) vs. time following HFS in different experimental groups. Bar graphs show the average fEPSP slope change (%) during 60 min post-HFS. Data are expressed as means ± SEM % of baseline. *P < 0.05

**Fig. 6:**
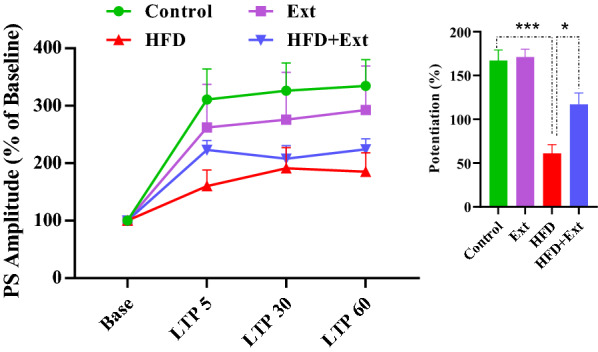
Time-dependent changes in hippocampal responses to perforant path stimulation following HFS. LTP of the PS amplitude in area DG granular cell synapses of the hippocampus are significantly different between groups. Left panel shows PS amplitude change (%) vs. time following HFS in different experimental groups. Bar graphs show the average PS amplitude change (%) during 60 min post-HFS. Data are expressed as means ± SEM % of baseline. *P < 0.05, ***P < 0.001

Field potential recordings were obtained in the granular cells in the DG following stimulation of the PP. Representative example of evoked field potential in the DG recorded prior to and 60 min after high-frequency stimulation is shown in Fig. 4. The effects of *R. damascena* extract on the EPSP slopes and PS amplitudes of rats fed with high-fat diet are shown in Figs. 5 and 6, respectively. We found that HFS did not induce LTP in rats fed with high-fat diet [F (3, 24) = 0.3207, P = 0.8103, one-way ANOVA]. The percent change in slope of fEPSP immediately and 60 min after HFS was significantly smaller in rats fed with high-fat diet than in control rats. We used a two-way analysis of variance to reveal the variability between the groups. Our results showed a significant effect of time points [F (3, 57) = 26.94, P < 0.0001] and treatment [F (3, 19) = 3.390, P = 0.0393] in slope of EPSP of the granular cell of DG (Fig. 5). Our post hoc analysis indicated significant differences between the control group and the HFD animals. Slope of EPSP decreased in the HFD group respect to control group (P < 0.05, Fig. 5). *R. damascena* extract consumption in the HFD group enhanced EPSP slope of the granular cell of DG (P < 0.05, Fig. 5). The percent change in slope of fEPSP immediately and 60 min after HFS was significantly greater in HFD + Ext group than in HFD group.

### Effects of *R. damascena* extract on the PS amplitude of granular cells in the DG of rats fed with high-fat diet

Our results showed a significant effect of time points [F (3, 57) = 29.84, P < 0.0001] and treatment [F (3, 19) = 8.497, P = 0.0218] in PS amplitude of the granular cell of DG (Fig. 6). Our post hoc analysis indicated significant differences between the control group and the HFD animals (P < 0.05, Fig. 6). PS amplitude decreased in the HFD group with respect to control group. *R. damascena* extract consumption in the HFD group enhanced PS amplitude of the granular cell of DG (P < 0.05, Fig. 6).

## Discussion

This study assessed the influence of the administration of *R. damascena* on in vivo hippocampal LTP in the PP-DG pathway in adult rats fed with an HFD. In the present study, we showed that the HFD impaired LTP induction in the PP-DG synapses. This conclusion is supported by decreased EPSP slope and PS amplitude of LTP. Therefore, we confirmed the observations of previous studies, in which HFD leads to synaptic plasticity impairment. Here, for the first time, we successfully identified that *R. damascena* supplementation prevents the destructive changes induced by HFD in hippocampal synaptic plasticity. Moreover, consumption of HFD decreased TTG and SOD in compare with control animals. *R. damascena* extract consumption in the HFD group enhanced TTG and SOD.

Herbal remedies with lipid lowering and antioxidant as well as anti-inflammatory activities can play a beneficial role for the management of the HFD-induced alteration of synaptic plasticity and brain glucose metabolism [60, 65]. HFD brains elicited decreased LTP relative to the control group. This HFD-induced alteration of synaptic plasticity could be a result of (a) the inability of the now insulin-resistant hippocampal neurons to generate enough neurotransmitter glutamate [66]; (b) impaired insulin signaling (Insulin receptor substrate (IRS) and Ras/Raf/ERK signaling nodes) [66, 67]; (c) decreased translocation of glucose transporters (GLUT3/GLUT4), creating a deficiency in energy substrates required for neurotransmitter production. [66, 67]; (d) reduced activation of the extracellular signal-regulated kinase/ cAMP-response element-binding protein (ERK/CREB) pathway [67]; (e) decreased the brain-derived neurotrophic factor (BDNF) expression and downstream mRNA levels for CREB and synapsin I [68]; (f) reduction of dendritic arborization and decreased dendritic spine number, and increase in reactive astrocytes [6].

LTP elevates the post-synaptic density of AMPA receptors. Insulin modulates glutamatergic neurotransmission by inducing GluR2 subunit phosphorylation in the AMPA receptor in the hippocampus, leading to endocytosis and thus decreases the post-synaptic excitatory ability [69].

In our study, the positive effects of *R. damascena* extract on hippocampal LTP in the PP- DG pathway in the HFD + extract group were likely due to its antioxidant properties. *R. damascena* extract consumption in the HFD animals decreased oxidative stress. Also, in our work, total phenolic and flavonoid contents of *R. damascena* were 3110.10 ± 10.20 and 1240 ± 14.5 mg per 100 g of extract, respectively. Flavonoids have antioxidant effects associated with various diseases, such as cancer, AD, etc. [70, 71]. Their high antioxidant capacity could be a result of following mechanisms [70]: (a) direct scavenging of ROS; (b) activation of antioxidant enzymes; (c) metal chelating activity; (d) reduction of α-tocopheryl radicals; (e) inhibition of oxidases; (f) mitigation of oxidative stress caused by nitric oxide; (g) increase in uric acid levels; (h) increase in antioxidant properties of low molecular antioxidants. It is reported that citronellol, geraniol, linalool, kaempferol and quercetin are the main components of *R*.* damascena* [26, 50]. These compounds have antioxidant properties [72]. It has also been shown that *R*.* damascena* provides protection against DNA oxidative damage through its significant antioxidant effects [73].

Emerging evidence suggests that flavonoids are able to activate signalling pathways, which are critical in controlling synaptic plasticity [74]. Their ability to activate the ERK1/2 and the protein kinase B (PKB/Akt) signalling pathways, leading to the activation of the CREB. These molecular events, which converge on CREB activation and neurotrophin synthesis, are able to induce synaptic plasticity [74]. In addition to ERK and CREB activation, flavonoids results in an activation of mechanistic target of rapamycin (mTOR) and an increased expression of hippocampal activity-regulated cytoskeleton-associated protein (Arc) [75]. Arc is known to be important in LTP and has been proposed to be under regulatory control of both BDNF [76] and the ERK signaling [77]. Also, flavonoids lead to improvements in synaptic plasticity through induction of synapse growth and connectivity, increases in synaptic activity, increases in dendritic spine density and the functional integration of old and new neurons [74]. Flavonoids are also capable of influencing neurogenesis through the activation of PI3 kinase-Akt-eNOS [78]. On the other hand, HFD has a detrimental effects on neurogenesis and neural plasticity in the hippocampus [67, 79], which appears to be masked by flavonoids or compounds containing flavonoids [78].

## Conclusion

In conclusion, the present study clearly demonstrates that treatment with the *R. damascena* can prevent synaptic plasticity impairment caused by the consumption of an HFD. These effects are likely due to the strong antioxidant properties of the extract and its ability to scavenge free radicals. Further experiments are required for determining the detail mechanism(s) of *R. damascena* action.

## Data Availability

All data and material are available.
